# Assessment of COVID-19 severity levels and associated factors among patients admitted to the treatment centers in Southern Ethiopia

**DOI:** 10.3389/fmed.2024.1403615

**Published:** 2024-10-31

**Authors:** Lire Lemma Tirore, Mihretu Tagesse Sergindo, Abriham Shiferaw Areba, Aklilu Habte Hailegebireal, Mitiku Desalegn

**Affiliations:** ^1^School of Public Health, College of Medicine and Health Sciences, Wachemo University, Hosaina, Ethiopia; ^2^Faculty of Health and Environmental Sciences, Auckland University of Technology, Auckland, New Zealand; ^3^Department of Anesthesia, College of Medicine and Health Sciences, Wachemo University, Hosaina, Ethiopia

**Keywords:** COVID-19, severity levels, ordinal logistic regression, Southern Ethiopia, associated factors

## Abstract

**Background:**

COVID-19, a highly impactful infectious disease, has been observed to result in psychological distress, organ impairment, and mortality. The severity and consequences of the illness appear to vary based on geographical location and individual characteristics. Understanding the disease and optimizing resource distribution through early classification depend on having data on the severity of COVID-19 patients. There is a dearth of information in this particular region regarding the severity of COVID-19 patients and related factors. Therefore, this study used an ordinal logistic regression model to determine the severity levels of COVID-19 and its associated components.

**Materials and methods:**

A retrospective follow-up study was conducted on COVID-19 patients admitted between May 30, 2020, and October 15, 2021, at care centers in southern Ethiopia. 845 patients were included in this research. The mean (standard deviation) and median (interquartile range) were used to summarize the data. A multivariable ordinal logistic regression model was used to study the association between independent variables and COVID-19 severity levels.

**Results:**

In terms of the severity of the disease, 12.07% of patients had severe COVID-19, 7.81% had critical disease, and 6.39% had moderate disease. 8.28% of the 845 patients died, while 88.88% of them made a full recovery. Older age (> = 40 years) (AOR = 5.75, 95% CI = 3.99, 8.27), comorbidities (AOR = 4.17, 95% CI = 3.03, 5.88), and low oxygen saturation (AOR = 3.44, 95% CI = 2.23, 5.56) were significantly linked to higher odds of experiencing more severe levels of COVID-19 compared to their counterparts.

**Conclusion:**

7.81% of patients were critically ill, while more than one-tenth (12.07%) were considered severely ill. Low oxygen saturation, comorbidities, and advanced age were found to be significantly associated with COVID-19 severity. Therefore, it is crucial to manage comorbidities, provide special treatment, and provide COVID-19 patients with underlying medical issues more attention due to the higher risk of poor outcomes. To speed up their recovery, medical professionals should regularly monitor and provide specialized care to older COVID-19 patients. In order to identify patients who are more likely to experience a severe illness and to better manage their treatment, it is imperative that oxygen saturation levels in COVID-19 patients be promptly identified and monitored.

## 1 Introduction

Severe acute respiratory syndrome coronavirus 2 (SARS-CoV-2) is the cause of the global coronavirus disease-2019 (COVID-19) pandemic of 2019. In Wuhan, in the Hubei province of China, a new coronavirus known as SARS-CoV-2 was discovered to be the cause of COVID-19 in December 2019 (1, 2). The primary route of entry for coronavirus infections is the respiratory system in humans. The main target of coronavirus infection is the human respiratory system (3).

Individuals with COVID-19 can present in a variety of ways, such as being asymptomatic, experiencing typical symptoms like fever, headache, dry cough, and breathing difficulties, or presenting with atypical symptoms like hyposmia, nasal congestion, rhinorrhea, sputum, abdominal pain, vomiting, and diarrhea (4). There are four severity classifications for COVID-19: mild, moderate, severe, and critical illness (5).

The global spread of SARS-CoV-2, despite control measures (6), caused a pandemic to be declared by the World Health Organization (WHO) on March 11, 2020 (7). As of 24 July 2022, over 567 million confirmed cases and over 6.3 million deaths have been reported globally. By September 22, more than 12 million COVID-19 cases had been reported across African Union Member States (8).

Individual differences can affect how the illness progresses, resulting in a range of outcomes, from quick recovery to multi-organ dysfunction and finally, death (9–11). As a result of it, five out of every 100 patients pass away (8). The prolonged hospital stays and high resource use linked to each COVID-19 patient can result in considerable financial losses for hospitals, which means that the economic impact of COVID-19 could be dire (12).

Predictors of COVID-19 disease severity include age, gender, smoking history, pre-existing medical disorders, body temperature, respiratory rate, oxygen saturation at admission, body mass index (BMI), and abnormal laboratory results (1, 9, 13).

On March 13, 2020, the first confirmed case of COVID-19 in Ethiopia was identified. The Ethiopian government has implemented a number of public health interventions in response to the COVID-19 crisis, including raising public awareness, closing schools, restricting large gatherings and mobility, and setting up treatment facilities with the necessary equipment to care for COVID-19 patients and quarantine anyone in contact with confirmed cases (8). The COVID-19 pandemic is spreading quickly and unexpectedly throughout Ethiopia and the world, putting pressure on hospitals, intensive care units (ICUs), and other healthcare resources in spite of the previously indicated protective measures.

Studying COVID-19 severity in Southern Ethiopia contributes to a more nuanced understanding of the pandemic and guides effective and context-specific public health responses. Knowing how severe COVID-19 is in this area can aid with the development of localized public health plans and interventions. This covers immunization campaigns, healthcare infrastructure development, and resource allocation. Researching the severity of the illness can shed light on how COVID-19 spreads among various populations, especially in underserved or rural locations like southern Ethiopia, and can also assist discover risk factors unique to the area.

Due to its low resources, southern Ethiopia may encounter particular healthcare issues. Examining the severity of COVID-19 can aid in determining how prepared and able the healthcare system is to handle spikes in instances. The severity of COVID-19 may be significantly impacted by socioeconomic factors, such as public health messaging, healthcare access, and education. For focused interventions, it is essential to comprehend these processes.

Insights gained from studying COVID-19 can inform responses to future outbreaks, whether they be of COVID-19 variants or other infectious diseases. This helps build resilience in public health systems.

Early detection of indicators of serious illness at presentation may help medical staff decide which patients are best managed in nearby hospitals and which ones need to be transferred right away to specialist institutions. Thorough information regarding the severity levels of COVID-19 patients is essential for improving our knowledge of the illness and possibly maximizing resource use by facilitating early prognostic classification. The identification of risk variables associated with the severity of COVID-19 may facilitate the timely identification of high-risk patients who require vigilant supervision, proactive supportive care, and timely intervention. Finding trustworthy indicators of the severity of an illness is essential to improving outcomes and preserving medical resources. Additionally, it assists in forecasting the future needs for ICU beds, ventilators, and necessary personnel. Early differentiation between severe and non-severe patients using basic clinical criteria may contribute to lowering mortality rates. In the study area, nothing is currently known about the COVID-19 severity levels and associated factors. Therefore, using an ordinal logistic regression model, the goal of this study is to assess the COVID-19 severity levels and associated factors.

## 2 Materials and methods

### 2.1 Study setting and period

The study was carried out in the Southern Nations, Nationalities, and Peoples’ Region (SNNPR) at the COVID-19 Treatment Centers. SNNPR, which makes up more than 10% of the country’s total area, is the third-largest administrative region in the nation and is renowned for its linguistic, cultural, and ethnic variety. The region is home to over 80 different ethnic groups, and Hawassa, the capital, is situated 273 km south of Addis Ababa. Kenya to the south, South Sudan to the west, Gambela to the northwest, and Oromia to the north and east are the borders of the SNNPR. The region had 17 COVID-19 care centers. The study was conducted between May 30, 2020, and October 15, 2021 (14).

Study design: A facility-based retrospective follow-up study was conducted.
Study population: all patients admitted to COVID-19 treatment institutions in the Southern Nations, Nationalities, and Peoples’ Region (SNNPR) between May 30, 2020, and October 15, 2021, who tested positive for the virus using rRT-PCR, were the study population. Patient cards with insufficient details about important characteristics such as the date of admission, the date of discharge, and the status of discharge were not included in the analysis.
Sampling technique: cluster sampling was employed. The SNNPR contains seventeen treatment centers (TC). Initially, the ideal cluster sample was determined using the rule of thumb as below:

$$n=\sqrt{\frac{\mathrm{total}\;\mathrm{number}\;\mathrm{of}\;\mathrm{cluster}}{2}}=\sqrt{\frac{17=}{2}}\sqrt{8.5}\;=3\;\mathrm{(Rule of Thumb)}$$

After that, four TC—the Nigist Elleni Mohammed Memorial Comprehensive Specialized Hospital, Agana Primary Hospital, Worabe Comprehensive Specialized Hospital, and Otona Comprehensive Specialized Hospital—were chosen at random. All patients (845) who were admitted to these treatment centers were included consecutively.
Data collection tool and procedure: The data collection tool was developed after reviewing the existing literatures to identify the important variables to be included in the tool. Also the patient charts and COVID-19 registration log book were observed to establish the trend of registration, physical examination, and history taking. Certain factors from follow-up, discharge, and patient registration records were combined with pertinent literature to construct a data extraction tool. The tool includes sections for clinical and sociodemographic data. Competent medical staff with treatment center experience handled the data extraction procedure (15–17).

Using secondary data from patient records might lead to sampling bias because the data may not fully represent the entire population. Additionally, inconsistent charting in medical records—often caused by time constraints, human error, variations in documentation practices, or incomplete data entry—can result in missing or inaccurate information. This can lead to measurement bias, which may affect the accuracy of the results.

### 2.2 Data quality assurance

Supervisors and data collectors received instruction on how to use the data extraction tool, its contents, and the consequences of poor data quality. Supervisors corrected mistakes, guided data collectors, and kept an eye on the gathering of completed checklists. EpiData was used for data entry and modification, and the data collectors received timely feedback. To find outliers in continuous variables, box plots were used.

### 2.3 Study variables

Outcome variables: severity levels of COVID-19 (1. asymptomatic, 2. mild, 3. moderate, 4. severe, 5. critical).
Independent variables include oxygen saturation, age, sex, residence location, TC, and the existence of co-morbidities.

### 2.4 Operational definition

Asymptomatic patients are those who tested positive for COVID-19 but do not show any symptoms.

Affected people who meet the criteria for the COVID-19 case description but do not show signs of hypoxia or pneumonia are considered mild cases. Common symptoms include fever, coughing, tiredness, anorexia, dyspnea, and myalgia. Other nonspecific symptoms include headaches, nausea, vomiting, diarrhea, runny noses, conjunctivitis, sore throats, nasal congestion, and loss of taste or smell.

Adolescents or adolescents who exhibit fever, cough, dyspnea, and rapid breathing along with clinical indicators of pneumonia but do not exhibit severe symptoms, such as blood oxygen saturation levels ≥90% at room temperature, are classified as moderate cases.

Severe cases are defined as adolescents or adults who exhibit clinical indicators of pneumonia (fever, cough, dyspnea, and rapid breathing) together with one of the following symptoms: a respiratory rate greater than 30 breaths per minute, severe respiratory distress, or oxygen saturation lower than 90%. When a child exhibits coughing or breathing problems along with at least one of the following pneumonia symptoms, it is deemed a severe case: lower than 90% oxygen saturation or central cyanosis, significant respiratory distress, including rapid breathing, grunting, and significant chest indrawing, as well as general warning indicators such as the inability to swallow or nurse, unconsciousness or lethargy, or convulsions.

The presence of sepsis, septic shock, acute respiratory distress syndrome (ARDS), or other complications such as acute pulmonary embolism, acute coronary syndrome, acute stroke, or delirium characterizes critical cases (18).

#### 2.4.1 Data analysis

Stata software version 15 was used to analyze the data. Frequencies and percentages were used to summarize the categorical variables. Depending on the kind of variable, tables, text, and graphs were used to present the results. The mean with standard deviation was used for continuous variables that were normally distributed, while the median with interquartile range was used for variables that were not normally distributed. Because the outcome variable was divided into ordered categories—(1) Asymptomatic, (2) Mild, (3) Moderate, (4) Severe, and (5) Critical—an ordinal logistic regression (OLR) model was used. Bivariate analysis was performed using proportional odds model to identify which variables had association with the severity level. All covariates which had an association with the outcome variables at *p*-value of 0.25 or less were entered into the multivariable model. This approach was used to ensure that important variables are not missed or overlooked in the multivariable analysis. The independent effects of covariates on odds of severity levels were analyzed using multivariable proportional odds model. Adjusted odds ratios with 95% Confidence Interval (CI) were estimated and *p* value less than 0.05 was used to declare presence of significant association between severity level and covariates. The ordinal logistic regression (proportional odds) model can be written in terms of the cumulative logits as:

The ordinal logistic regression (proportional odds) model can be written in terms of the cumulative logits as:


(1) $$\log\;\left[\frac{\mathrm{Pic}}{1-\mathrm{Pic}}\right]=\gamma c-\left(x_{i}\beta \right)$$

Pic is a cumulative probability of being in the “c” category of COVID-19 for *i*th individual. **_𝛄_c** is a model threshold or intercept for the C-1 level of COVID-19.

It represents the cumulative logits of being at or below the C-1 level of COVID-19 when the covariates are equal to zero. It is strictly increasing (i.e., γ1 < γ2 < … < γC − 1).

C = number of categories of COVID-19 which equals 5. *β* is a coefficient (fixed effect of explanatory variable).

*X_i_* is a covariate vector for *i*th individual.

#### 2.4.2 Assessing model assumption

Proportional odds assumption (POM): The assumption of proportional odds states that the effects of all independent variables remain consistent across all levels of the dependent variable. This assumption was evaluated by the Brant test.

## 3 Results

The study involved 845 patients admitted to selected healthcare facilities in southern Ethiopia from May 30, 2020, to October 15, 2021. Around two-thirds, 566 (66.98%) of the patients being male. The participants’ median age was 30 years (interquartile range = 25 years). The majority of participants, 391 (46.27%) were from OCSH TC (Table 1).

**Table 1:** Socio-demographic characteristics of COVID-19 cases admitted to treatment centers of Southern Ethiopia, 2021.

Variables	Category	Severity levels					Total (%)
		Asymptomatic	Mild	Mode rate	Severe	Critical	
Residence	Rural	261	100	35	70	56	522 (61.78)
	Urban	214	48	19	32	10	323 (38.22)
Age	≤25	192	44	14	5	2	257 (30.41)
	26–40	193	40	10	21	14	278 (32.90)
	≥41	90	64	30	76	50	310 (36.69)
Sex	Male	315	93	33	71	54	566 (66.98)
	Female	160	55	21	31	12	279 (33.02)
Treatment center	OCSH	291	64	15	9	12	391 (46.27)
	Agana primary hospital	141	30	8	39	3	221 (26.15)
	WCSH	8	52	31	18	32	141 (16.69)
	NEMMCSH	35	2	0	36	19	92 (10.89)

### 3.1 Baseline clinical characteristics of patients

More than one-fifth of the patients (192, 22.72%) had a comorbidity, while 83 patients (9.82%) had low oxygen saturation; 2.72% needed mechanical breathing; and 5.44% were supported by nasal oxygen. Table 2 shows that 56.21% of the patients had no symptoms. The most common comorbidity was hypertension (4.26%), which was followed by diabetes mellitus (3.55%).

**Table 2:** Clinical characteristics of COVID-19 cases admitted to treatment centers of Southern Ethiopia from May 30, 2020 to October 15, 2021.

Variables	Category	Severity level					Total (%)
		Asymptomatic	Mild	Moderate	Severe	Critical	
Comorbidity	Yes	50	30	20	48	44	192 (22.72)
	No	425	118	34	54	22	653 (77.28)
Oxygen saturation	Normal	463	139	48	61	51	762 (90.18)
	Poor	12	9	6	41	15	83 (9.82)
Outcome	Recovered	459	134	51	73	34	751 (88.88)
	Died	11	7	1	21	31	23 (2.71)
	Referred	5	7	2	8	1	70 (8.28)

Comorbidity: hypertension, DM, HIV, and TB. This table does not differentiate between patients with just one comorbidity (e.g., hypertension) and those with two or more comorbidities (e.g., hypertension, diabetes, and HIV). As a result, it combines all patients with any comorbidity into a single category, without distinguishing the potential impact of multiple comorbidities on severity, oxygen saturation, and outcomes.

### 3.2 Severity levels among COVID-19 patients

**Figure 1:**
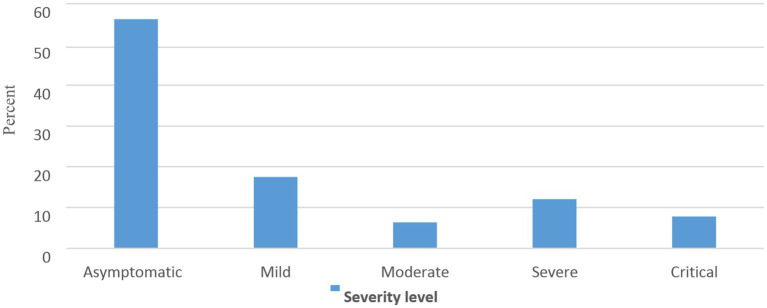
Percentage distribution of severity levels among COVID-19 cases admitted to care centers of Southern Ethiopia, 2022.

Concerning the severity of the disease, 17.51% had mild disease and 12.07% had severe disease (Figure 1).

### 3.3 Factors associated with the severity level of COVID-19

In the initial bivariate ordinal regression analysis, covariates with a *p* value <0.25, such as age, comorbidity, and oxygen saturation, were selected for inclusion in the multivariable analysis. In the multivariable analysis, age, comorbidity, and oxygen saturation were found to be significantly associated with different severity levels of COVID-19. The proportional odds assumption was met for these significant variables. The odds ratios for the significant variables were applied and interpreted by comparing higher severity levels of COVID-19 with lower levels (e.g., critical versus severe and below, critical and severe versus moderate and below, moderate and above versus mild and asymptomatic, and mild and above versus asymptomatic COVID-19). This approach was possible because the proportional odds assumption held for the significant variables.

COVID-19 patients aged 40 years and above were 5.75 times more likely to experience higher severity levels of COVID-19 compared to younger patients (aged 25 years and below) (AOR = 5.75, 95% CI = 3.99, 8.27). Patients with comorbidities were 4.17 times more likely to face higher severity levels than those without comorbidities (AOR = 4.17, 95% CI = 3.03, 5.88). Individuals with poor oxygen saturation had 3.44 times higher odds of experiencing a higher severity level of COVID-19 compared to those with normal oxygen saturation (AOR = 3.44, 95% CI = 2.23–5.56) (Table 3).

**Table 3:** Factors associated with severity levels of COVID-19 among patients admitted to treatment centers of Southern Ethiopia from May 30, 2020 to October 15, 2021.

Variables	Category	COR (95%CI)	AOR (95%CI)	*p* value
Residence	Urban	0.23 (0.10, 1.50)		0.53
	Rural	1		
Age	≤25	1	1	
	26–40	0.96 (0.65, 0.98)	1.40 (0.95, 2.06)	0.08
	≥41	0.68 (0.4, 0.85)	5.75 (3.99, 8.27)	0.0001*
Sex	Male	1		
	Female	1.35 (0.72, 1.05)		0.67
Treatment Center	OCSH	0.85 (0.23, 1.86)		0.81
	Agana primary hospital	1.32 (0.5, 2.59)		0.56
	WCSH	1		
	NEMMCSH	0.65 (0.32, 2.89)		0.87
Comorbidity	Yes	4.10 (1.32, 5.26)	4.16 (3.03, 5.88)	0.0001*
	No	1	1	
Oxygen saturation	Poor	4.12 (1.21, 6.33)	3.44 (2.23, 5.56)	0.0001*
	Normal	1	1	

OCSH, Otona Comprehensive Specialized Hospital; NEMMCSH, Nigist Elleni Mohammed Memmorial Comprehensive Specialized hospital; WCSH: Worabe Comprehensive Specialized Hospital; Comorbidity: Hypertension, DM, HIV, and TB. *p value < 0.05; COR, Crude odds ratio; AOR, Adjusted odds Ratio; CI, Confidence Interval.

Health authorities can create targeted interventions by having a better understanding of the elements that contribute to the severity of COVID-19. This implies that resources can be focused on high-risk groups, guaranteeing that treatments and preventative measures are given priority to those who are most likely to suffer from serious consequences.

Tracking factors associated with severity can enhance epidemiological monitoring and modeling of disease spread. Health authorities can adjust their strategies based on real-time data to respond more effectively to outbreaks. Policymakers can use insights derived from the analysis of severity factors to craft policies that address higher-risk groups, such as prioritizing vaccination for those at greater risk or implementing localized lockdowns in areas with high transmission and severe outcomes. In general, knowing the COVID-19 severity factors aids in navigating the pandemic’s intricacies and enables local health strategies to be more effective and nuanced while making the most use of the resources at hand to safeguard the public’s health.

Asymptomatic or moderate cases may go unnoticed and unadmitted, providing an insufficient understanding of the COVID-19 pandemic. Because secondary data may not fully represent the total population, using it could lead to sample bias. Moreover, irregular charting or missing data could have led to measurement bias, which would have undermined the reliability of the findings.

## 4 Discussion

The main goal of this study was to describe the severity of COVID-19 and the factors associated with it in patients who were being treated at southern Ethiopian medical facilities. The study found that the percentages of mild, moderate, severe, and critical cases of COVID-19 were 17.51, 6.39, 12.07, and 7.81%, respectively. These numbers show that the prevalence of severity levels is lower than in a previous study that was carried out at the Ethiopian Millennium COVID-19 care center, where the rates of moderate and severe cases were much higher (32.07 and 32.65%, respectively) (19) and at the at the Fifth Affiliated Hospital of Sun Yat-sen University (Zhuhai, China) (35%). The disparities found in the research may be explained by differences in the prevalence of comorbidity and demographics among the populations. The severity of COVID-19 can be greatly impacted by variations in population factors, including age, sex, ethnicity, and underlying medical disorders. Variations in public health interventions, such vaccination campaigns, social distancing rules, and mask laws, might affect the pace of transmission and, ultimately, the severity of the disease in a particular place. It is possible that areas with stricter public health regulations report lower severity levels.

Patient outcomes may be impacted by the capability and resources of regional healthcare systems, including the accessibility of medical facilities, tools, and medical staff. The methods used to quantify and report severity may vary depending on testing rates, hospitalization criteria, and reporting procedures.

The Fifth Affiliated Hospital of Sun Yat-sen University (40%) and the Millennium COVID-19 Care Center (38.9%) had significantly higher rates of comorbidity than the study’s findings.

Comorbidity, advanced age, and low oxygen saturation were found to be associated with the severity of the COVID-19 infection. Compared to younger patients, older patients were more likely to have higher severity levels of COVID-19. This result is in line with the Millennium COVID-19 care center’s findings (19), Ethiopia, Zagazig University, Egypt (20), and Premier Hospital, Kenya (15). The higher risk of severe illness among elder COVID-19 patients may be explained by aging-related declines in the immune system, mitochondrial function, and hormone levels, as well as multimorbidity and poor nutrition in the elderly. It becomes harder for people’s bodies to fend off diseases like the coronavirus as they get older since their immune systems tend to deteriorate. Advanced age increases the risk of developing COVID-19-related consequences since older people are more likely to have underlying medical illnesses such as diabetes, heart disease, and respiratory problems. Furthermore, elderly individuals can react to the virus more slowly than younger people, which would allow it to multiply and do more harm before the immune system can establish a strong fight (16, 21). However, age was not associated with COVID-19 severity in a study done at Ascension St. John Hospital, Michigan (22), and the Fifth Affiliated Hospital of Sun Yat-Sen University (23). This discrepancy might be due to the difference in the statistical technique used, sample size, and age categorization. In a study done in Michigan, age was categorized as <60 and > 60 years, which is different from this study.

Compared to individuals without comorbidity, those with comorbidity had a higher likelihood of having COVID-19 disease at a higher severity level. Research from the Millennium COVID-19 treatment centers in Ethiopia (20), Ascension St. John Hospital, Michigan (22), and Montichiari Hospital, Italy (17), supports this conclusion. This may be because innate and adaptive immunity malfunction as a result of chronic illness, raising the likelihood of severe COVID-19 disease and making the body more vulnerable to other diseases that compromise the immune system. Chronic inflammation is linked to a number of comorbidities, which can worsen the body’s reaction to the virus and cause more severe symptoms. Individuals who have multiple medical conditions may have lowered physiological reserves, which can make it more challenging for their systems to handle the additional strain of a serious viral infection (24). Contrary to this, comorbidity was not associated with severity in studies done at Zagazig University, Egypt (20) and the Fifth Affiliated Hospital of Sun Yat-sen University (Zhuhai, China) (23). Disagreement across studies may be due to variations in patient status and the quality of healthcare provided in each setting.

Compared to patients with normal oxygen saturation, those with poor oxygen saturation were more likely to be at a higher severity level of COVID-19. This result is consistent with that of Sun Yat-sen University’s Fifth Affiliated Hospital (Zhuhai, China) (23), Ascension St. John Hospital, Michigan (22), and Benjamin G. et al., 2021 (25). This may be due to the fact that lower oxygen saturation is a sign of a sick lung with reduced capacity, which is also explained by the SARS-CoV-2 virus’s strong lung-attacking affinity. However, a study conducted at Zagazig University in Egypt found no correlation between oxygen saturation and the severity of COVID-19 disease (20).

Ensuring high-risk groups, including the elderly and those with comorbidities, receive timely vaccinations and booster doses can prevent the severe disease among these group of population. Also implementing routine screening for high-risk individuals to detect early symptoms and potential complications of COVID-19 could prevent the severe disease. Developing targeted campaigns to educate high-risk groups about the importance of COVID-19 precautions, vaccination, and recognizing symptoms early is another recommendation to reduce the severity of the COVID-19.

Strengths of this study include utilizing modern statistical approaches, taking into account many centers, and having a large sample size. Asymptomatic and/or mild cased may have been overlooked. Using secondary data from patient records might lead to sampling bias because the data may not fully represent the entire population. Additionally, inconsistent charting in medical records—often caused by time constraints, human error, variations in documentation practices, or incomplete data entry—can result in missing or inaccurate information. This can lead to measurement bias, which may affect the accuracy of the results. The reliance on hospital admissions may have introduced sampling bias, as more severe cases are likely to be hospitalized, skewing results toward higher severity levels. Controlling for certain confounders, such as laboratory indicators, becomes challenging when using secondary data. The comorbidity data were verbal reports, which may not accurately reflect the patients’ true conditions.

## 5 Conclusion

7.81% of patients were critically ill, while more than one-tenth (12.07%) were considered severely ill. Low oxygen saturation, comorbidities, and advanced age were found to be significantly associated with COVID-19 severity. Therefore, it is crucial to manage comorbidities, provide special treatment, and provide COVID-19 patients with underlying medical issues more attention due to the higher risk of poor outcomes. To speed up their recovery, medical professionals should regularly monitor and provide specialized care to older COVID-19 patients. In order to identify patients who are more likely to experience a severe illness and to better manage their treatment, it is imperative that oxygen saturation levels in COVID-19 patients be promptly identified and monitored.

## Data Availability

The raw data supporting the conclusions of this article will be made available by the authors, without undue reservation.
